# Risk of falling among elderly persons living in the community: assessment by the Timed up and go test

**DOI:** 10.5935/1808-8694.20130004

**Published:** 2015-10-14

**Authors:** Onivaldo Bretan, José Elias Silva, Odilon R. Ribeiro, José Eduardo Corrente

**Affiliations:** Adjunct Professor (Adjunct Professor in the Otorhinolaryngology Course in the Department of Ophthalmology, Otorhinolaryngology, and Head and Neck Surgery at the Medical School at Botucatu, UNESP); Undergraduate Student (Undergraduate Student at the Medical School at Botucatu, UNESP); Adjunct Professor (Adjunct Professor in the Department of Biostatstcs at the Insttute of Biosciences at Botucatu, UNESP). Medical School at Botucatu - UNESP

**Keywords:** assessment, balance, elderly, fall

## Abstract

The risk of falling in elderly can be analyzed by a simple mobility test.

**Objective:**

To assess the balance of elderly subjects through the 'Timed Up and Go' test.

**Method:**

Subjects were timed for the moment they got up from a chair, walked for three meters, and came back to the chair. They also answered questions on imbalance, dizziness, and falls.

**Results:**

Approximately 69% of the subjects completed the test in up to 19 seconds. There was a significant correlation between imbalance, time spent in the test, dizziness, and falls.

**Conclusion:**

Most of the elderly subjects performed well in the test, thus attesting to their good level of functional mobility. However, a significant number of poor-performers is probably more prone to falling and to depending on others to perform activities of daily living.

## INTRODUCTION

The risk of falls in the elderly has been investigated through questionnaires, clinical balance an gait tests, force platforms, and posturography^1^, ^2^, ^3^, ^4^, ^5^, ^6^. Clinical tests such as the Berg Balance Scale, the Tinetti Mobility Test, and the Timed Up and Go (TUG) test have been extensively used. Unlike the TUG, the first two take longer and require more examiner training^1^, ^2^, ^3^. The TUG test targets diseased elderly subjects, and has been used to investigate specific diseases, physical and mental conditions, complaints of imbalance and falls, subjects belonging to certain age ranges and genders, and has been applied in case-control studies^3^^,^^6^, ^7^, ^8^, ^9^, ^10^, ^11^. Enrollment and exclusion criteria vary significantly in the literature, as do reference TUG test results for healthy elderly subjects^10^^,^^12^^,^^13^. Systemic disease in the elderly has been closely correlated with imbalance and falls^14^^,^^15^. The TUG test can realistically assess mobility and balance in the elderly by scoring one's risk of falling in connection with the activities of standing up, walking, turning around, and sitting down^3^^,^^10^.

There are no studies in the literature on the performance of community-dwelling elderly in the TUG test that explore criteria other than the ones that allow subjects to undergo the test, i.e., ability to understand commands and walk without assistance. Such a study would facilitate the verification of the actual status of balance in this population. This study aimed to assess the balance of elderly living in the community through the Timed Up and Go test.

## METHOD

This descriptive cross-sectional study enrolled elderly subjects of both genders, seen at the Geriatric Care Ward of a Local Health Care Center (*Centro de Saúde Escola*). A group of 102 individuals was tested, 66% of whom were females. Approximately 75% of the subjects were 75 and older (Table 1).

**Table 1:** Socio-demographic data (n = 102).

Females	67 (66%)
Aged 60-74 years	25.5%
Aged 75+ years	74.5%
Mean age	78 ± 7 years

Individuals unable to comprehend the situations of the test or walk without help, subjects with systemic disease, decompensated conditions or in a wheelchair were excluded. We tried to stick as much as possible to what the authors of the test did, despite the few restrictions in their exclusion criteria - many of their subjects were stroke patients and had other neurological diseases - which dictated that the 'medically unstable' and patients with grade IV Parkinson's could not be enrolled^3^.

The patients were briefly interviewed to establish the diseases they had been diagnosed with that could make them prone to falls, and the symptoms suggestive of increased risk of falling. The subjects were asked about the number of times they had fallen within the last six months^2^^,^^5^, ^6^, ^7^. Diseases and disorders were referred to by their more colloquial equivalents, as advised by the subjects' geriatrician. The elderly and their guardians were enquired about heart, kidney, and lung disease, myocardial infarction and stroke. They were asked about possible sequelae of stroke that could directly or indirectly compromise balance such as vertigo, gait disorders, loss of muscle strength, ataxia, and sensory symptoms of the face and chest consistent with nerve injury caused by occlusion of the territories irrigated by the vertebrobasilar system^16^.

The answers provided a good overview of the physical conditions that could turn patients more prone to falls. The complaints of dizziness refer to an unspecific term used by the patients to describe sensations of vertigo, often referred to as ‘labyrinthitis,' and to include loss of consciousness (syncope) or the symptoms preceding it (pre-syncope) in the form of blacked out or blurred eyesight, instability, imbalance, weakness, lightheaded-ness, falls, etc.^16^. Complaints of imbalance referred to objective alterations in static or dynamic posture. This study was approved by the institution's Research Ethics Committee (permit 4132/2012).

The TUG test was applied in accordance with the definitions set by its authors^3^. Subjects seated on an armchair were asked to stand up and walk to a line on the floor, turn around, and sit down on the armchair again. They were timed from the moment the examiner said ‘go.' Times under 10 seconds are suggestive of completely free and independent individuals; subjects with times between 10 and 19 seconds are independent and possess reasonable balance and gait speed, and most can walk for over 500 meters, climb stairs, and go out by themselves. Patients taking 20 to 29 seconds are in a ‘grey area,' i.e., they have varying difficulties in performing activities of daily living depending on the situations they are in, which may require good balance, proper gait speed (of at least 0.5 m/s), and functional capacity^3^. Times of 30 seconds or more tend to reflect subjects who are highly dependent on others to perform basic activities of daily living (getting up from a chair, feeding, changing, bathing, walking). The subjects were previously shown what the test consisted of and were instructed to walk at their usual pace and speed. In case of doubt from the subjects of the examiner, the test was repeated. For safety reasons, the examiner walked right by the patients during the test.

## RESULTS

Table 2 describes the incidence rates of the various reported diseases and complaints. A significant number of patients had high blood pressure, dyslipidemia, and complained of imbalance. Table 3 presents the patients' times and scores. Four (3.81%) of the 102 individuals spent under 10 seconds to finish the test. Sixty-five individuals (63.7%) took 10 to 19 seconds, and 17 (16.76%) spent 20 to 29 seconds. Sixteen subjects (15.80%) took 30 or more seconds to finish the test. Age and time spent, number of falls and age, and number of falls and time spent on the test were not significantly correlated. Complaint of ‘dizziness,' and number of falls (*p* = 0.03), complaint of ‘imbalance,' and number of falls (*p* = 0.02), and complaint of ‘imbalance,' and time spent on the test (*p* < 0.0001) were statistically correlated. Complaint of dizziness *vs.* number of falls corrected for age was statistically significant, with age being an independent factor for dizziness. Imbalance *vs.* time spent on the test corrected for age was not statistically significant, i.e., age is a confounding factor for imbalance. Imbalance *vs.* number of falls corrected for age was statistically significant, i.e., age is an independent factor for imbalance.

**Table 2:** Clinical data.

Reported diseases and complaints	${\text{N}}^{\underline{\text{o}}}$ of individuals	Percent
Diabetes*	21	20.5%
High blood pressure*	74	72%
High cholesterol*	43	42%
Heart disease*	27	26%
Infarction	12	11.7%
Stroke	16	15.5%
Kidney disease*	7	6.8%
Lung disease*	7	6.8%
Dizziness*	34	33%
Labyrinthitis*	31	30%
Imbalance*	51	50%
Two or more falls within the last six months*	25	24%

* Current diseases and complaints.

**Table 3:** Time spent on the TUG test.

Time (seconds)	${\text{N}}^{\underline{\text{o}}}$ of individuals
< 10	4 (3.81%)
10 to 19	65 (63.72%)
20 to 29	17 (16.76%)
≥ 30	16 (15.80)

Mean: 19 ± 12 seconds.

## DISCUSSION

Seventy percent of the subjects spent 19 seconds or under to complete the test. Individuals performing at this level present few sporadic mobility limitations while carrying out activities of daily living^3^. Thirty percent of the individuals took 20 or more seconds to finish the test. This includes both individuals with maximum mobility restrictions, i.e., people who are highly dependent on others even while at home, and subjects with varying risks of falls for activities performed in and out of their homes^3^. Scores of 20 seconds and above are clearly worse than the times observed in studies done on populations of normal elderly subjects, whose times ranged between 11 and 12 seconds^10^^,^^12^^,^^13^. According to Podsiadlo & Richardson^3^, when compared to normal reference values, even part of the subjects deemed independent in the 10-to-19-second group have poor scores. The systemic diseases present in the subjects in our study may explain the observed time differences, given that they may compromise one's balance^11^^,^^13^^,^^14^^,^^17^^,^^18^.

Incidentally, approximately 31% and 34% of them respectively reported ‘labyrinthitis,' and dizziness, while 51% and 25% reported imbalance and falls respectively. Metabolic syndrome is known to affect 30% to 50% of the Brazilian elderly, and hyperglycemia, dyslipidemia, and high blood pressure are associated with syncope and ves-tibulopathy^11^^,^^17^, ^18^, ^19^, ^20^. In spite of what our study found, other authors have analyzed normal populations and shown that performance on the TUG test worsens with age^12^^,^^13^. The small size of our series, and possibly the fact that 75% of our population was made of people 75 and older, may have prevented the occurrence of a significant correlation. Age did not interfere with worse performance on the test or with imbalance reported by the subjects, but had an impact on the number of falls in subjects with complaints of dizziness and on the number of falls of individuals complaining of imbalance. No papers on diseased elderly were found to compare subjects for age and TUG test scores.

Complaint of imbalance *vs.* number of falls and imbalance *vs.* more time spent on the test were statistically correlated, as also supported by the literature. However, complaint of dizziness, although associated with more falls, was not statistically correlated with lower TUG test scores corrected for age^6^, ^7^, ^8^, ^9^.

The results of this study have shown significant differences when compared to other normative and reference studies, proving that the TUG test is a good instrument to assess the risk of falls^8^^,^^10^, ^11^, ^12^, ^13^. Other studies support this statement, and have shown significant differences between non-fallers and subjects who have fallen two or more times within six months^6^^,^^7^. Reference and normative values must be seen from a qualitative standpoint, i.e., they allow the exclusion of subjects not at risk of falling from fall prevention protocols. On the other hand, the TUG test's quantitative scores may be useful in clinically deciding how to tackle the patients' risk of falls. This does not mean that they should be taken as the absolute truth, once the samples of tested elderly subjects vary significantly in terms of the diseases they have, the medications they take, and their physical statuses^6^^,^^13^. This test may be seen as a screening tool to be used at busy basic health care centers and administered by nurses, physicians, physical therapists or other health care professionals after some training^3^.

The limitations of this study do not allow the generalization of its results. These limitations stem from sample size and the fact that the patients enrolled came from a group of goers of a geriatric care center, and not the elderly population in general. A population study would be a more adequate way to obtain scores subject to more general application and portray the status of balance among the elderly. Such a study requires organization and a qualified working team. It is worthwhile mentioning that population studies inevitably include healthy and diseased subjects, both treated and untreated.

## CONCLUSION

Most of the elderly subjects in this study were at a low risk of falling, as indicated by their good functional mobility. However, a significant number of individuals had worse scores in the TUG test and were probably more prone to falls, in addition to facing more limitations while performing activities of daily living.
